# The Comparative Oncology of Canine Malignant Melanoma in Targeted Therapy: A Systematic Review of *In Vitro* Experiments and Animal Model Reports

**DOI:** 10.3390/ijms251910387

**Published:** 2024-09-26

**Authors:** Xiaohui He, Yu Gao, Yuqing Deng, Junying He, Ingo Nolte, Hugo Murua Escobar, Feng Yu

**Affiliations:** 1 Department of Small Animal Medicine, College of Veterinary Medicine, China Agriculture University, Beijing 100193, China; 2 Department of Small Animal Medicine and Surgery, University of Veterinary Medicine Hannover, Foundation, 30559 Hannover, Germany; 3 Department of Medicine, Clinic III, Hematology, Oncology and Palliative Medicine, University Medical Center Rostock, 18057 Rostock, Germany

**Keywords:** melanoma, canine, target therapy, immunotherapy, protease inhibitor, microRNA

## Abstract

Canine malignant melanoma (CMM) is highly aggressive and mostly located in the oral cavity. CMM is the predominant type of canine oral malignancy and shows striking homologies with human mucosal melanoma. In comparative oncology, canine oral melanomas (COMs), as spontaneous tumor models, have the potential to acquire a unique value as a translational model of rare human melanoma subtypes. This review aims to provide a comprehensive summary of targeted therapies for canine malignant melanoma and to enrich the field of comparative oncology. Following the PRISMA guidelines, a comprehensive literature search was conducted across databases for studies from 1976 to April 2024. Studies were selected based on their relevance to targeted treatments. A total of 30 studies met the inclusion criteria. Based on the treatment approaches, the studies were further categorized into immunotherapies, small molecule signaling inhibitors, indirect kinase inhibitors, and other alternative strategies. Some treatments have been shown to result in stable disease or partial response, accounting for 29% (monoclonal antibody) and 76.5% (micro-RNA therapies) in clinical trials. Moreover, in vitro experiments of small molecule inhibitors, including cell signaling inhibitors and indirect kinase inhibitors, have shown the potential to be an effective treatment option for the development of therapeutic strategies in canine malignant melanoma. The observed response in in vitro experiments of CMM (particularly the oral and certain cutaneous subtypes) to drugs used in the treatment of human melanoma underlines the resemblance to human melanoma, therefore supporting the notion that CMM may be a valuable model for understanding rare human melanoma subtypes and exploring potential therapeutic avenues in preclinical trials. Finally, this literature review serves as a valuable resource for the development of therapeutic strategies for CMM and highlights the potential for translating these findings to human cancer treatment.

## 1. Introduction

Canine melanoma causes the abnormal proliferation of melanocytes and can occur in the oral cavity (44–62%), haired skin (27–44%), digits (8–10%), and eyes (1–3%) of any dog breed [1]. It accounts for approximately 4–7% of all canine malignant tumors, and oral melanoma accounts for 30–40% of all canine oral tumors [1,2]. Most melanomas are pigmented and appear black due to a high melanin content, but non-pigmented melanomas (amelanotic melanomas) also exist. The prognosis of canine melanoma depends on the location of the tumor. Canine melanoma of the haired skin tends to behave in a less malignant manner, while canine mucosal melanoma is recognized for its aggressive nature, high rate of metastasis, and potential for local invasion [3,4].

Melanoma affects both humans and canines, with severe clinical outcomes. In comparative oncology, canine melanoma, particularly the mucosal variant, emerges as a compelling model for human melanoma due to its similar histological features, biological behavior, and genetic alterations [5]. In 2012, the National Cancer Institute Comparative Melanoma Tumor Board compared the histological features of canine oral melanoma (COM) and canine melanomas arising in other sites (skin and acral) with human malignant melanoma (hMM) and canine malignant melanoma (CMM), finding a complete concordance between COMs and hMMs [3]. Additionally, the unique position of canine melanoma as a model for human melanoma stems from several shared characteristics between the two. Both canine and human melanomas are often aggressive and resistant to conventional therapies, presenting challenges in management and treatment [3,4]. The activation of the same molecular pathways involved in canine and human melanoma progression, such as alterations in the Mitogen-Activated Protein Kinase (MAPK) and phosphatidyl-inositol 3-kinase/serine-threonine kinase (PI3K/AKT) pathways, show remarkable similarity across species. However, ultraviolet (UV) irradiation triggers mutagenesis, and *BRAF* (v-raf murine sarcoma viral oncogene homolog B1) has high frequency hotspot mutation in human melanoma but is not characteristic of canine melanoma. Therefore, canine melanoma models can further validate the use of canine models in translational research [4].

In recent years, there have been significant advances in the field of targeted therapies for canine melanoma, reflecting a shift toward more individualized approaches to medicine [6,7]. Innovations in immunotherapy [8] and small molecule inhibitors [9] offer new perspectives for prolonging survival and improving the quality of life for dogs with melanoma. Targeted therapy is a new and developing modality that disrupts dysregulated pathways unique to cancer cell biology, thereby reducing systemic side effects. As the understanding of the molecular landscape of canine melanoma deepens, targeted therapies emerge as a beacon of hope for improving treatment outcomes [10,11].

This review examines various human-targeted therapies that have been employed in the treatment of canine melanoma, including an analysis of their underlying mechanisms, their efficacy, and the insights they provide into the management of melanoma in canines. In particular, the application of therapies targeting specific molecular pathways, immune checkpoint inhibitors, and personalized medicine approaches in canine melanoma offers insights into their potential effectiveness and challenges in human clinical settings. This review integrates findings from veterinary and human oncology to systematically assess the current landscape of targeted therapies and their clinical efficacy. It also highlights the significant contributions of comparative oncology to the field of melanoma research. A comprehensive analysis of in vitro studies, animal model research, and clinical trials is presented to provide an up-to-date synthesis of the therapeutic landscape for canine melanoma and highlight promising strategies that may pave the way for breakthroughs in the management of melanoma. In this way, we also highlight the similarities between melanoma in humans and dogs, emphasizing the advantages of a synergistic approach to drug development and therapeutic strategies in advancing cancer research and treatment across species.

## 2. Materials and Methods

### 2.1. Literature Search

Based on the Preferred Reporting Items for Systematic Reviews and Meta-Analyses (PRISMA) framework, a comprehensive and systematic literature review was conducted using the sites PubMed (https://pubmed.ncbi.nlm.nih.gov/ accessed on 1 May 2024) and Web of Science (http://apps.webofknowledge.com/) (accessed on 1 May 2024). For the title or abstract search, the search MESH terms included [melanoma], [canine, dog], [target, molecule, inhibit], [therapy, experiment], [*in vitro*, *in vivo*] for OR searching in each term set, and each term set was used together for AND searching, to identify relevant references. Only papers published between 1976 and April 2024 were included.

### 2.2. Inclusion and Exclusion Criteria

The inclusion criteria were as follows: (1) original research articles about targeted therapy for canine melanoma; (2) the article was written in English; (3) in vitro or animal model research or clinical trial; and (4) detailed information was present, including types of target treatments, mode of application and dosage of the agent, animal model and/or cell line, response rate, and anti-tumor effect. Excluded criteria were as follows: (1) the article was not published in English; (2) the article type was review/systemic review article, patent, letter, conference abstract/paper; (3) only an abstract was available; (4) study object was not specifically detailed for canine melanoma.

### 2.3. Data Collection

First, the articles obtained from the database search were screened by their abstract for relevance. Subsequently, the full text of the studies selected in the second stage of screening was analyzed to assess whether the inclusion criteria were satisfied. The whole search procedure and all study selections were performed by three authors (X.H., Y.D., J.H.) to determine suitability for inclusion in this review. For immunotherapy, information was extracted by category, immunotherapeutic strategy, study subjects, study outcome, and references. For small molecule inhibitors, proteases/kinases targeted therapy and other targeted therapies, the information extracted included inhibitor and mechanism, study subject, response rate, and references. 

### 2.4. Assessment of Risk of Bias in Included Studies

Among these total 30 studies, 16 were only included in vitro experiments, 2 were only included in vivo experiments, 5 were included in both in vitro and in vivo experiments, and 7 were reports of clinical trials. The assessment of risk of bias was based on the methodological index for non-randomized studies (MINORS) measure [12]. The MINORS measure contains and sets an eight-item evaluation for non-comparative studies and a twelve-item evaluation for comparative studies (for summary, see Supplement Table S1), with a maximum score of 16 and 24, sequentially, for the corresponding assessment of the two kinds of studies. For the reason that the included literature was too heterogeneous, a meta-analysis was not available for the selected studies. And since the results of each study cannot be directly compared, an overall assessment of the quality across studies was not performed. The MINORS (Methodological Index for Non-Randomized Studies) scoring system is designed to assess the methodological quality of non-randomized studies. Although it is primarily used to evaluate clinical studies, it can also be appropriately applied to animal studies and other non-randomized biomedical studies, provided that these study designs include comparable groups. For experimental studies using animal models and cell lines, the scoring excludes the criterion of “inclusion of consecutive patients”, which typically pertains to clinical studies that do not enroll consecutive patients, so the total score was 14.

## 3. Results

### 3.1. Search Results

**Figure 1:**
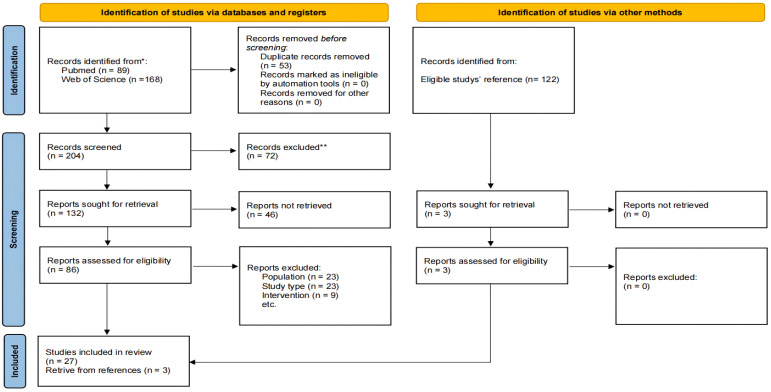
PRISMA flow diagram for inclusion of studies in the structured literature review. * The number of records identified from each database. ** According to the language type (not in English), article type (review or conference) or the unavailable access to full text.

From the total of 257 studies that were initially screened for inclusion, 27 fulfilled the criteria for enrolment. Moreover, we scrutinized the citations within the eligible publications for relevant studies; only 3 out of 122 papers surveyed met the criteria and were included in this review. The flow of information is shown in the PRISMA diagram (Figure 1). Among these total 30 studies, 16 only included in vitro experiments, 2 only included in vivo experiments; 5 included both in vitro and in vivo experiments, and 7 were reports of clinical trials. The eligible studies included 47 cell lines in total (Table 1). These studies were described and discussed in the following categories: immunotherapies (n = 5); small molecule signaling inhibitors (n = 10); indirect kinase inhibitors (n = 7); other alternative treatments, including engineering target drug systems (n = 3); microRNA therapies (n = 3); old drug, new use (n = 2).

This series of studies primarily focuses on treatment methodologies for canine melanoma, especially the exploration of novel drugs and their mechanisms of action against this disease. The research encompasses the evaluation of the therapeutic effects of various drugs, targeted therapies, and immunotherapies on canine melanoma cell lines and actual cases. The mechanisms of these treatments often involve the inhibition of cell proliferation and migration capabilities, as well as promoting cancer cell apoptosis by activating specific cell death pathways. Additionally, these studies explore enhancing the immune system’s ability to recognize and eliminate tumor cells by targeting specific cell surface molecules or signaling pathways.

### 3.2. Immune Checkpoint Inhibitors and Their Role in Melanoma Treatment

CMM is a spontaneously occurring aggressive tumor with relatively few medical treatment options, which provides a suitable model for the disease in humans. Historically, multiple immunotherapeutic strategies aimed at provoking both innate and adaptive anti-tumor immune responses have been published, with varying levels of activity against canines [25]. Dogs have a complex immune system, similar to that of humans, and live in similar environments; due to their similarities, modern immunotherapies in human medicine have the potential to be translated into treatments for canine melanoma. Since monoclonal antibodies (mAbs) targeting specific cancer antigens have been successful in mediating immune responses against tumors in humans [26], this treatment has been gradually adopted by veterinary medicine into clinical application. Gangliosides GD2 and GD3 are expressed in various types of human tumors and were identified in canine OMM through immunohistochemical analysis. Hence, anti-ganglioside mAbs were developed and implicated in the treatment of canine oral malignant melanoma combined with human recombinant interleukin-2 (IL-2). While IL-2 and mAbs have been extensively used in human medicine, their application in veterinary medicine, particularly for canine melanoma, is innovative and effective in vitro [27,28]. The mAbs can specifically recognize gangliosides on canine melanoma cells and enhance tumor cell lysis through antibody-dependent cell-mediated cytotoxicity (ADCC), which demonstrates the success of translative research in canine melanoma. 

Checkpoint inhibitors have transformed the treatment landscape for melanoma; they not only have led to significant improvements in survival for various types of tumors in humans [29,30] but also have provided an applicable option for canine melanoma. Led by anti-Podoplanin mAbs, canine chimeric anti-PD-L1 mAbs (c4G12), and anti-PD-1 mAbs, studies in veterinary medicine also employ different immune-checkpoint inhibitors towards into clinical trials. The ratio of cases in immune-checkpoint inhibitors achieving a partial response was three out of 28 cases (anti-PD-1 and PD-L1 mAbs) and stable disease in six out of 24 cases (anti-podoplanin and anti-PD-1 mAbs). These therapeutic approaches have demonstrated an objective response rate in pilot clinical trials for the treatment of canine oral malignant melanoma [31,32,33]. However, in human melanoma, PD-1 and PD-L1 inhibitors have shown response rates ranging from 15% to 40% [34]. In these studies, PD-L1 expression plays a significant role in the response to immune-checkpoint inhibitors in canine OMM, similar to observations in human melanoma. However, due to the lack of comprehensive PD-L1 expression data in these canine studies, a direct comparison is challenging. Therefore, matching expression levels and further research on PD-L1 as a biomarker in dogs could potentially lead to better response rates.

Among the aforementioned strategies, another innovative treatment is anti-HER2 chimeric antigen receptor tumor-infiltrating lymphocytes (CAR-TILs) that are genetically modified to more effectively recognize and attack tumor cells. This represents a significant advancement in both human and canine tumors, as it overcomes resistance by employing a patient’s own immune cells. Recently, the use of CAR-T cell therapy has yielded remarkable success in the treatment of specific types of human cancers, particularly those of the blood and lymphatic systems, such as acute lymphoblastic leukemia. While in human solid tumors, including melanoma, CAR-T cell therapy has shown significantly lower response rates compared to hematologic malignancies [35,36]. Forsberg et al. conducted a FIDO (First-in-Dog) trial to provide safety data for a planned first human trial. The study showed that CAR-TILs therapy showed promising results in a canine melanoma model. CAR-TILs therapy has achieved a tumor-free state in one subungual melanoma with metastasis to the prescapular lymph node. Another metastatic oral malignant melanoma experienced a reduction in tumor size, though the disease eventually progressed and the canine was euthanized [37]. The success in canine models justifies further research and the initiation of first-in-human trials focusing on similar genetic modifications to direct the immune system against cancer cells, particularly in solid tumors like melanoma.

In conclusion, the exploration of immunotherapies for canine melanoma offers a compelling glimpse into the parallel advances in veterinary and human oncology. From the strategic use of monoclonal antibodies targeting gangliosides GD2 and GD3 to the innovative implementation of immune checkpoint inhibitors and CAR-TIL therapies, the spectrum of efficacy demonstrates the complex nature of cancer treatment. Each therapeutic approach, while promising, demonstrates variability in outcomes (see Table 2), which highlights the nuanced challenges of effectively combating melanoma.

### 3.3. Cell Signaling Inhibitors

Canine melanomas share a similar dysregulation with human melanomas in the MAPK (mitogen-activated protein kinase) and PI3K/AKT (phosphoinositide 3-kinase/protein kinase B) pathways [38,39], which are crucial in cell proliferation, survival, and melanoma pathogenesis. Targeted therapy for melanoma mainly targets the MAPK/ERK and PI3K/AKT/mTOR pathways, the main objective is to inhibit tumor growth and to identify resistance mechanisms.

*In vivo* studies investigated the mTOR (mammalian target of rapamycin) inhibitor rapamycin, aiming at the PI3K/AKT pathway, showing a significant impact on cell growth and survival in a dose-dependent manner [40]. At the concentration of 10nM, rapamycin significantly reduced the phosphorylation of mTOR and p70S6K (target of the mTOR pathway), which showed the potential to inhibit key survival signaling pathways [13]. Meanwhile, combination therapies targeting different molecular targets have shown to be more effective, potentially leading to better clinical outcomes with lower dosages and overcoming resistance to single-agent therapies. For example, AZD6244 (MEK inhibitor) and rapamycin can effectively target the MAPK and PI3K/AKT signaling pathways, reducing the phosphorylation levels of ERK1/2 and p70S6K, with IC_50_ values between 5.7 and 391 nM [38]. 

Trametinib has been approved by the FDA for the treatment of human melanoma with *BRAF V600E* or *V600K* mutations and has shown efficacy in inhibiting tumor growth and improving survival in patients with *BRAF* mutant melanoma [41]. The combination of trametinib and sapanitib (mTORC1/2 inhibitor) can effectively reduce the viability of melanoma cells and shows a good anti-tumor effect both in vivo and *in vitro*. Sapanisertib is better tolerated than dactolisib [22], and the combination of sapanisertib and trametinib can effectively inhibit tumor growth and metastasis, achieving the purpose of higher efficacy and lower toxicity [42]. The drug effectively inhibited the growth of six canine melanoma cell lines at concentrations ranging from 0.1 to 5 µM and induced early and late apoptosis/necrosis, indicating its potential as a promising therapeutic agent for treating melanoma in canines and providing insights for human clinical applications.

The advancements in the treatment of canine melanoma not only improve outcomes for dogs but also provide valuable insights into human melanoma treatment, particularly in the fields of comparative oncology and translational medicine. Gao et al. emphasized the potential of using canine melanoma as a model to study human melanoma by investigating the application of the inhibitor LY3009120 in the canine, human, and equine malignant melanoma cell lines [24]. LY3009120 targets multiple *RAF* family members, enabling a comprehensive blockade of the MAPK pathway and overcoming upstream resistance. LY3009120 effectively inhibited the growth of six canine melanoma cell lines at concentrations ranging from 0.1 to 5 µM and induced early and late apoptosis/necrosis in canine melanoma cell lines. This suggests its potential to be a promising therapeutic agent in inhibiting cell proliferation and promoting the death of melanoma cells. 

Rivoeranib is a tyrosine kinase inhibitor that selectively inhibits VEGFR2, which can effectively inhibit canine melanoma in vitro and *in vivo*; it inhibits proliferation and migration, induces cell cycle arrest and apoptosis, and down-regulates VEGFR2 phosphorylation and cyclin-D1 expression [43,44]. Another tyrosine kinase inhibitor, toceranib, had a transient therapeutic effect on a canine case of OMM carrying a *KIT c.1725_1733del* mutation, suggesting that specific gene profiles can influence the response to targeted therapies [45]. While *KIT* mutations in canine melanoma are not as common as in other cancers like canine mast cell tumors [46], their identification can critically inform treatment decisions and highlight potential therapeutic targets. Toceranib and rivoceranib both showed benefits but may be more relevant to cases with specific genetic profiles.

Some studies focus on different targets, identifying and exploiting specific weaknesses in cancer cells to develop more effective and less toxic treatments for canine melanoma. MLN4924 can target the NEDD8-regulated neddylation system, significantly reducing cell viability in a dose- and time-dependent manner, promoting apoptosis and inhibiting cell growth through the induction of DNA replication and senescence [47]. Some do not interfere with signaling pathways but are regulated by GSK-3β. 6-Bromoindirubin-3′-Oxime (BIO) enhances the transcriptional activity of β-catenin by inhibiting the activity of GSK-3β, thereby affecting multiple signaling pathways and inhibiting the proliferation, survival, and migration of tumor cells [18].

In conclusion, the shared pathogenic mechanisms between canine and human melanoma provide a foundation for the development of human melanoma treatments. Within many options for small signal inhibitors in canine melanoma (see Table 3), MEK inhibitors in combination with PI3K/mTOR inhibitors, such as trametinib and sapanisertib, show promise. However, the optimal treatment in the clinic depends on the genetic profile and pathway activation of the individual case, so it is essential to detect the mutational status of canine melanoma before implementing targeted therapies. As reported by studies, a shift is occurring towards individualized treatments and precision medicine, as the mutation landscape becomes transparent [7,11]. This shift is also reflected in small-molecule signaling inhibitors. It is becoming increasingly important to detect the mutational status of malignant melanoma in dogs before implementing targeted therapies, as this aids in identifying cases that may benefit from a specific targeted therapy and guide treatment decisions. 

### 3.4. Indirect Kinase Inhibitors

Instead of directly inhibiting kinase activity, many studies opened new pathways for the development of targeted therapies by interfering with other key biological processes that affect tumor growth and survival. This includes inhibiting molecular stability, altering gene expression, and interfering with signaling systems inside cells. Kuroki et al. screened 320 compounds and revealed that oligomycin, an F1Fo ATPase inhibitor, by regulating energy metabolism, exhibited potent growth-inhibitory effects on CMM cell lines while showing lesser toxicity on non-neoplastic control cell lines [16]. Some interfere with protein stability and functional regulation, such as ganetespib, an HSP90 inhibitor that is investigated in human clinical trials for various cancers, including lung and breast cancer [49]. A pioneering method was tested with STA-1474, a prodrug of ganetespib, showing in vivo efficacy in dogs with spontaneous cancers. Phase I clinical trial demonstrated a positive response in a dog with oral malignant melanoma after five treatment cycles, significantly reducing the tumor [50]. This unexpected outcome underscores STA-1474’s efficacy in targeting and mitigating cancer cell viability. 

Cancer cells show self-sufficiency in growth signals. Verdinexor (KPT-335), a selective inhibitor of nuclear export, can effectively inhibit the proliferation and induce apoptosis in canine melanoma cells in vitro by restoring the function of tumor suppressor proteins and interfering with signaling pathways that promote cell growth [23]. Further evaluation in preclinical and clinical settings in treating spontaneous cancers in dogs demonstrated the compound’s potential for a favorable safety profile and biological activity against the disease [15]. Together, these studies provide a solid scientific foundation for the application of verdinexor in treating canine melanoma, foreseeing its development as a novel therapeutic approach.

In terms of resistance mechanisms in aggressive tumors, some therapeutic approaches open new avenues for treatments. Histone demethylase inhibitors (HDIs) targeting JARID1B have shown promising results in canine melanoma cell lines and remain effective in cells resistant to cisplatin [51]. HDIs targeting the JARID1 family of histone demethylases were identified as highly expressed in canine tumor tissues. And JARID1 family is relevant to cell proliferation and drug resistance in human cancers [52]. The research HDIs, specifically PDCA (24-Pyridinedicarboxylic acid), can effectively reduce cell survival in melanoma cell lines without significantly affecting DNA damage repair kinetics, offering a potential treatment for oral melanomas that are resistant to conventional therapies [51]. 

Another novel strategy for canine melanoma is to inhibit the growth and spread of cancer cells by blocking the transport of certain essential amino acids required by the cancer cells. This is achieved by inhibiting LAT1, which is expressed at high levels in many tumors and low levels in normal tissues, making it an ideal target. LAT1 inhibitors like BCH and LPM prevent tumor spread while enhancing the effectiveness of other anticancer drugs [53].

The collective findings of the studies indicate that targeting different molecular pathways can be an effective strategy for treating canine melanoma. Detailed information is provided in Table 4. The most promising results seem to come from the novel orally bio-available XPO1 (selective inhibitor of nuclear export)-inhibitor KPT-335, which has demonstrated biological activity against canine melanoma cell lines at physiologically relevant doses. This drug also demonstrated the downregulation of the XPO1 protein, upregulation of the XPO1 message, increased expression of tumor suppressor proteins p53 and p21, and enhanced nuclear localization, indicative of direct effects on the intended target. In comparison, toceranib and rivoceranib also showed benefits but may be more suited to cases with specific genetic profiles, such as the presence of a *KIT* mutation for toceranib. However, there is no single “best” treatment, as the optimal approach likely depends on individual tumor genetics and behavior, the dog’s overall health, and other factors. These findings provide support for the further investigation and potential use of SINE compounds, with a particular focus on KPT-335, in the treatment of canine melanoma. 

### 3.5. Other Target Therapies

#### 3.5.1. Engineering Target Drug Systems

A wide range of alternative therapies exists, with the ideal scenario being drugs that target and accumulate specifically at tumor sites, effectively destroying tumor cells while sparing normal and stem cells with regenerative capabilities. Genetic engineering facilitates the modification of cells or therapeutic agents to enhance their anti-tumor effects and targeting abilities. For example, a study reengineered anthrax toxin, initially developed for human cancers, to target OMM by homing in on markers such as urokinase plasminogen activator (uPA) and metalloproteinases (MMP-2) [55,56]. This modified toxin was tested in a clinical trial involving five dogs with OMM, administered via intratumoral injections. Observations indicated that this approach stabilized the disease, reduced tumor volume, and induced necrosis within tumor cells and blood vessel walls, all without significant systemic side effects. Similarly, ICOCAV17, an oncolytic adenovirus adapted from human cancer therapies, targets a broad spectrum of canine cancers, including melanoma [57]. Eduardo Laborda et al.’s study explored the effects of this virus, engineered with an RGD (Arg-Gly-Asp) motif for enhanced infectivity and hyaluronidase for improved tumor penetration, on two canine melanoma cell lines [58]. Significant in vitro cytotoxicity and reduced tumor growth in in vivo xenograft models were observed. These targeted delivery mechanisms enable selective cancer cell destruction while minimizing damage to healthy cells, thereby reducing potential side effects.

One study incorporated genetic engineering techniques in canine adipose tissue-derived mesenchymal stem cells (cAT-MSCs), drawing upon strategies developed in human medicine [59]. Engineered cAT-MSCs were employed to produce IFN-β, which enabled the cells to home in on tumor sites and deliver the therapeutic agent directly [60]. cAT-MSC-IFN-β demonstrated significant inhibition of melanoma cell growth in vitro and reduced tumor volumes in vivo in a mouse xenograft model. It is noteworthy that when this approach is combined with conventional chemotherapy, IFN-β contributes to anti-tumor activity by inducing apoptosis and inhibiting cell proliferation, while cisplatin primarily causes DNA damage, leading to cell death. This dual strategy underscores the potential of combining gene therapies and chemotherapies for more effective melanoma treatment.

#### 3.5.2. MicroRNA Therapies

MicroRNAs (miRNAs) are small, non-coding RNAs that are 18-25 nucleotides long and highly conserved across species. [61]. Significant advancements have been reported in cancer therapy using miRNAs. One study investigated the intratumoral injection of synthetic miR-205 for canine melanoma, and the treatment yielded significant therapeutic outcomes in dogs at various stages of melanoma [62]. Following this, Yoshikawa’s team in 2023 investigated the anti-tumor effects of topical application of miR-634 on spontaneous CMMs [63]. Through in vitro experiments on CMM cell lines and in vivo trials in seven canine cases, they revealed that miR-634 induced apoptosis by downregulating Asct2, Nrf2, and surviving expression, observing anti-tumor effects in four cases without adverse reactions. Additionally, Wada et al.’s 2019 study explored the potential of miR-634 to enhance the radiosensitivity of canine oral melanoma cells, finding that miR-634 significantly reduced cell viability and promoted apoptosis in melanoma cells treated with radiation [64]. These studies collectively build a scientific foundation for employing miRNAs as a new strategy for treating canine melanoma, foreseeing its potential as a novel therapeutic approach for human malignant melanoma. miRNAs can be used as targeted therapeutic strategies and adjuncts to traditional treatments (such as surgery and radiotherapy) for COM. Particularly, the results from miR-205 and miR-634 research indicate that by modulating specific miRNAs, new therapeutic approaches can be developed, potentially improving the prognosis for COM treatment.

#### 3.5.3. Old Drug, New Use

Some studies show that NSAIDs (Nonsteroidal Anti-Inflammatory Drugs) may affect tumor cell survival, drug resistance, and other pathways, independent of their anti-inflammatory effects. One study that investigated NSAIDs’ effect on canine melanoma cell lines [20] suggested that the anti-tumor effects of NSAIDs are exerted through COX-independent pathways. Transcriptome analysis of a melanoma cell line treated with NSAIDs identified changes in the expression of several genes. Five genes (*SLC16A6*, *PER2*, *SLC9A8*, *HTR2B*, and *BRAF*) were commonly upregulated, while four genes (*LOC488305*, *H2AFJ*, *LOC476445*, and *ANKRD43*) were commonly downregulated. While NSAIDs exhibit anti-tumor activity in canine melanoma cell lines, the mechanisms of action appear to be independent of their traditional role in COX inhibition. The study identifies several candidate genes that may mediate these effects, but further research is required to fully understand the mechanisms and to explore the potential therapeutic applications of NSAIDs in cancer treatment.

Statins are a class of hydroxymethylglutarate monoacyl-CoA (HMG-CoA) reductase inhibitors that are often used as first-line drugs in human clinical practice to lower cholesterol levels and prevent cardiovascular and cerebrovascular disease [65]. In recent years, more and more studies have shown that statins can inhibit tumor cell proliferation, invasion, and metastasis, inhibit angiogenesis, promote tumor cell apoptosis, and synergistically enhance the effect of radiotherapy and chemotherapy [66]. Atorvastatin has the effect of inhibiting the growth of canine cancer cells with high ZEB (the epithelial-to-mesenchymal transition-inducing transcription factor) expression, especially the proliferation of mesenchymal-like cells. In an in vitro study that included three canine melanoma cell lines, atorvastatin significantly inhibited cell proliferation in the melanoma cell lines, showing high sensitivity to atorvastatin, with IC_50_ values ranging from 5.92 to 17.7 μM [21]. This indicates that these melanoma cell lines are highly responsive to atorvastatin, particularly in cells with high expression of the ZEB family of epithelial-to-mesenchymal transition (EMT)-inducing transcription factors, where the anti-proliferative effect of atorvastatin was more pronounced.

While each of these therapies has demonstrated varying degrees of efficacy (See Table 5), miRNA therapies present innovative and less conventional alternatives that have shown significant results and might be beneficial when traditional treatments are not suitable or as complementary treatments. The engineering of drugs and the repurposing of older drugs for novel purposes exemplify the adaptability and evolution of cancer therapy. This underscores the necessity for individualized treatment strategies based on the specific context of each case of canine melanoma.

## 4. Discussion

To the best of our knowledge, this comprehensive review systematically evaluates the effectiveness of various targeted therapies for canine melanoma. By scrutinizing the results from selected studies, our analysis categorizes these treatments based on their mechanisms of action and provides a detailed account of their efficacy in combating canine melanoma. Focusing on in vitro experiments and animal model reports, the review reveals a broad spectrum of therapeutic strategies being explored, including immunotherapies, small molecule signaling inhibitors, and alternative treatments such as engineered target treatments, microRNA applications, and the repurposing of existing drugs.

The review adheres to the PRISMA guidelines, facilitating a systematic and comprehensive literature search and selection process. Given the diverse range of study types and designs included in the literature, the applicability and accuracy of statistical analysis methods may be constrained. The study encompasses various types, including in vitro cell experiments, in vivo experiments with experimental animals, and case reports, which may result in heterogeneity in study outcomes. Additionally, the use of disparate methods for assessing results may introduce bias in the interpretation of results. There are 18 studies that only involve in vivo and/or in vitro studies of animal models and/or cell lines; thus, they are not applicable in terms of the “inclusion of consecutive patients”. However, in vitro studies typically account for all experimental samples, so there is no loss to follow-up in the traditional sense. The MINORS score of each included study can be seen in the Supplementary Materials, Table S2.

Maekawa et al. (2017) and Forsberg et al. (2023) conducted pilot clinical trials that were methodologically rigorous, with clear goals, prospective data collection, and well-defined endpoints such as tumor response rate and survival benefit. However, the small sample size and lack of a control group may affect the generalizability of the results [31,37]. Similar clinical trials by London et al. (2011) and Fukumoto et al. (2013) showed a high degree of methodological rigor [50,53], but Fukumoto et al. (2013) had no randomized control group or blind experimental setting, potentially overestimating treatment effects and possibly introducing performance and detection biases [53]. Fowles et al. (2015) and Yoshitake et al. (2017) conducted rigorous in vitro studies, including comprehensive analysis techniques such as detailed biochemical analysis, the use of control variables, and transcriptome analysis [20,38]. The main bias may stem from the limitations of in vitro models and their translation into clinical validity, as they do not fully reflect biological responses in humans or dogs.

Ahn et al. (2013) combined an in vitro model with an in vivo model to provide insights more likely to be relevant to a clinical setting by integrating gene therapy and chemotherapy in an animal model [60]. While in vitro studies provide important fundamental knowledge and molecular insights, the inclusion of in vivo studies provides a more comprehensive assessment of the therapeutic effects and potential clinical applications.

Overall, this article provides an overview of immune-targeted therapies for canine melanoma, with CAR-T cell therapy appearing particularly promising due to its potential to transform tumor reduction and maintain a tumor-free state in some cases. Nevertheless, its complexity and cost may impede its widespread use. Immune checkpoint inhibitors offer more broadly applicable options that can be readily integrated into existing treatment protocols. Pembrolizumab (humanized PD-1 inhibitor) and its canine equivalent PD-1 inhibitors have been used in both humans and dogs, with promising results that support their cross-species efficacy in the immune checkpoint blockade. However, the response rate shows that mAbs do not act as efficiently as in human melanoma. It cannot be denied that dogs and humans have intrinsic biological differences, including differences in immune system function and tumor biology. The efficiency of PD-1 and PD-L1 inhibitors in human melanoma is significantly influenced by the percentage of PD-1/PD-L1-positive cells. High expression levels of PD-L1 in tumor cells and PD-1 in TILs are associated with better therapeutic responses; thus, matching PD-1/PD-L1 expression levels in canine studies should be considered to improve response rates in dogs, as this has shown promising results in human studies [68]. Others hypothesize that TMB (tumor mutation burden) may also influence the response to immunotherapy. Higher TMB has been associated with a better response to immunotherapy in humans because it indicates a higher number of mutations, which may produce new antigens capable of initiating immune response [69]. In general, the lower TMB observed in canine melanoma compared to human cutaneous melanoma could be one reason for the generally less dramatic response to immune checkpoint inhibitors [70]. Further research and combination therapy may help improve the response rate to melanoma in dogs.

Kinase inhibitor therapies, particularly small molecule signaling inhibitors, have driven a shift toward more personalized treatments. They emphasize treatments that target specific mutations. Human melanomas are often driven by well-defined genetic mutations (e.g., *BRAF*, *NRAS*, and *KIT* mutations) that can be targeted by specific therapies. Most studies put forward that unlike human cutaneous melanomas, human and canine mucosal melanomas are more often associated with high copy number alterations, lower TMB, and structural variation burden rather than the high specific hotspot gene mutations (*BRAF* or *NRAS*) seen in cutaneous humans [7]. In contrast, some cases of mucosal melanomas in humans and canines have shared gene mutations, with *NRAS* mutations found in 12% (179/1454) of human cases and 9.6% (11/115) of canine cases [44,70]. Interestingly, targeted oncogenic signaling pathways were proven to be effective, even in cases where mutations in oncogenes occur at low frequencies or in wild-type (non-mutated) forms of canine melanoma. This implies that the effectiveness of the drugs may rely more on the general activity of these pathways rather than on specific mutations, highlighting a broader approach for therapeutic targeting in canine melanoma [38].

Beyond that, the emergence of innovative treatments such as the Pan-RAF inhibitor represents a broader strategy of inhibiting all *RAF* isoforms, thus addressing resistance issues that arise from selective RAF inhibitors. Amino acid inhibitors such as HDIs and L-Type Amino Acid Transporter 1 (LAT1) inhibitors show the potential for expanding the repertoire of effective therapies [71]. Histone demethylase inhibition opens a newer area of therapeutic development, which involves targeting epigenetic modifications that are crucial for maintaining the oncogenic phenotype in melanoma cells. LAT1 inhibitors block essential amino acid transport into melanoma cells, starving them of necessary nutrients and inhibiting their growth and survival [53].

The investigation of small molecule signaling inhibitors in canine melanoma is instructive for human medicine. Studies on canine melanoma not only contribute to veterinary medicine but also parallel advancements in human oncology. Rapamycin (Sirolimus), the mTOR inhibitor used in both humans and canines to inhibit cell proliferation in various cancers, including melanoma, has shown similar IC_50_ values in inhibiting melanoma cell growth in both species. Similarly, trametinib, the MEK inhibitor use in the treatment of human melanoma, especially those with *BRAF* mutations, has shown efficacy in trials, suggesting similar pathway inhibition. Imatinib is a TKI inhibitor primarily used in human chronic myeloid leukemia but also in gastrointestinal stromal tumors due to its inhibition of c-KIT, PDGFR, and ABL kinases. imatinib and toceranib target similar kinases and show comparable anti-proliferative effects in canine melanoma. NVP-BEZ235 (Dactolisib), a dual PI3K/mTOR inhibitor used in both human and canine studies, shows similar effectiveness in targeting the cellular pathways important in melanoma progression. A pan-RAF inhibitor (LY3009120) has been shown to inhibit both *RAS*^mut^ and wild type melanoma cell proliferation in vitro study and may have the potential for further applications in canine melanoma. Apatinib (Rivoceranib) (Advenchen Laboratories, California), initially developed for human use and later adapted for canines, is a VEGFR2 inhibitor that showed similar effects in reducing tumor vascularization and growth in melanoma. These studies provide crucial insights into how melanomas can be targeted based on specific pathway dysregulations, informing the development of precision medicine approaches in humans. By understanding how these inhibitors affect canine tumors, researchers can better design and optimize similar treatments for human patients. In selecting the optimal treatment plan, whether human or canine, it is imperative to take into account the specific type and stage of cancer. This is also objectively reflected in the 30 articles included. For instance, canine melanoma with specific genetic mutations, such as *NRAS* and *KIT*, may be particularly amenable to treatment with small molecule inhibitors that target these mutations. Canine melanoma with high expression of PD-L1 in the tumor micro-environment may be more suitable for the use of immune checkpoint inhibitors. In recent days, there have been some novel findings about the gut microbiome, which is crucial in regulating immune responses and has been shown to influence the immune response to melanoma, particularly in the context of immunotherapy. Certain microbial compositions can enhance the body’s ability to fight melanoma by improving the response to immune checkpoint inhibitors like anti-PD-1 and anti-CTLA-4 therapies, suggesting that onco-microbiotic therapies could be tailored to optimize treatment outcomes [72]. Dogs have environmental exposures and microbiome compositions similar to those of humans; they can serve as a bridge in understanding how microbiome alterations might affect melanoma treatment in humans [73]. Future research should continue to leverage the strengths of comparative oncology, not only improve therapeutic outcomes for canine patients but also to refine and expand treatment options for human melanoma. By continuing to align canine and human research, we can enhance our understanding of melanoma at a molecular level, improve the predictive value of preclinical models, and accelerate the development of novel therapeutics for both species.

However, some limitations exist since many of the studies rely on in vitro experiments and animal models, which may not fully replicate the complex interactions and responses observed in clinical settings with actual canine patients. Consequently, the strength of evidence available to support the efficacy of these treatments is limited due to the absence of randomized controlled trials (RCTs) among the reviewed studies.

## 5. Conclusions

In conclusion, the study establishes a foundation for future research directions, with the aim of improving treatment outcomes and enhancing the quality of life for canine patients with melanoma. The investigation of targeted therapies for canine melanoma offers a distinctive perspective in the field of comparative oncology, facilitating the integration of insights between canine and human cancer treatments. Canine melanoma serves as a valuable model for human melanoma due to the similarity in histological and genetic characteristics and therapeutic response, which facilitates the translation of findings across species. This review underscores the value of comparative oncology in advancing cancer research and treatment strategies across species. Notably, therapies such as immune checkpoint inhibitors and small molecule signaling inhibitors, which have demonstrated efficacy in human medicine, are now being adapted for use in canine melanoma, with potentially promising results. These adaptations not only enhance treatment outcomes for dogs but also provide critical data that can influence human cancer treatment protocols. This systematic review also highlights the necessity for detailed genetic and molecular profiling prior to the application of targeted therapies, whether canine or human.

## Figures and Tables

**Table 1:** The summary of cell lines used in the included studies.

Cell Line	Origin	Molecular Mutation Type	Ref.
12	Primary tumors of COM	Not mentioned	[13]
23	Primary tumors of COM		
50	Primary tumors of COM		
17CM98	Lymph node metastasis of COM	Not mentioned	[14]
323610-3	Not mentioned	*BRAF* ^WT^	[15]
ChMC	COM	Not mentioned	[16]
CMeC-1	Primary canine skin melanoma	Not mentioned	[17]
CMeC-2	CMeC-1 Xenotransplantation lung metastasis		
CML-1	Primary COM	Not mentioned	[18]
CML-2	Primary COM		
CML-10C2	Canine cutaneous melanoma		
CML-6M	Lymph node metastasis of COM		
CMM-1	Primary oral melanoma from canine with lymph node metastasis	Not mentioned	[19]
CMM-2	Primary COM with no metastasis		
CMM7	Not mentioned	Not mentioned	[20]
CMM8	Not mentioned		
CMM9	Not mentioned		
CMM10	Not mentioned		
CMM11	Not mentioned		
CMM12	Not mentioned		
HTR	Not mentioned	Not mentioned	[21]
ITP	Not mentioned		
KMeC	Primary canine oral melanoma	Not mentioned	[17]
LMeC	Lymph node metastasis of COM		
NIP	COM	Not mentioned	[16]
NML	Canine nail bed melanoma		
OMJ	COM	Not mentioned	
PuMeC	Not mentioned	Not mentioned	[21]
UCDK9M1	Skin metastasis of COM	*NRAS* ^WT^	[22]
UCDK9M2	Lymph node metastasis COM	*NRAS* ^WT^	
UCDK9M3	Primary COM	*NRAS* ^WT^	
UCDK9M4	Primary COM	*NRAS* ^WT^	
UCDK9M5	Lymph node metastasis of COM	*NRAS* Q61 mutation	
Mel 23	primary COM	Not mentioned	[23]
Mel 36	Metastatic lymph node		
Mel 69	primary COM		
Mel 83	primary COM		
YCC	Not mentioned	Not mentioned	[21]
Jones	Primary tumor, COM	*NRAS* Q61 mutation	[22]
Parks	Primary tumor, COM	Not mentioned	
cRGO1	Primary tumor, COM	*BRAF*^WT^, *KRAS*^WT^, *NRAS*^G13R^	[24]
cRGO1.2	Lymph node metastasis of cRGO1 primary tumor, resistant to irradiation (0–6 Gy)		
cRGO4	Primary tumor, COM	*BRAF*^WT^, *NRAS*^WT^, *KRAS*^WT^	
cRGO6	Cutaneous metastasis of COM		
Mel1268	Primary tumor, COM		
Mel0910	Primary cutaneous malignant melanoma		
TLM1	Primary tumor, COM	Not mentioned	[10]

COM: canine oral melanoma; WT: wild type; *NRAS* Q61 mutation: changing in the *NRAS* gene at the codon for the amino acid glutamine (Q) located at position 61; *NRAS*^G13R^: the substitution of glycine (G) with arginine (R) at the 13th position of the NRAS protein.

**Table 2:** The efficacy of different immunotherapy modalities in canine melanoma.

Immunotherapeutic Strategy	Study Subject	Study Outcome	Ref.
Anti-ganglioside monoclonal antibodies: Murine Mab 14.G2a and its mouse-human chimera	*In vitro* experiment CML-2, CML-6M	CML-6M: mAbs 14.G2a (anti-GD2) and R24 (anti-GD3) mediate ADCC against the cell line. Combination with IL-2 enhanced the cytotoxicity. CML-2: no significant expression of GD2 or GD3, and hence, it was not responsive to the antibodies.	[27]
Immune Checkpoint Inhibition: Canine chimeric monoclonal antibody	A pilot clinical trial of seven dogs with OMM: stage II × 1; stage III × 2; stage IV × 4	PR: 1/7 (stage II × 1) PD: 6/7 (stage III × 2; stage IV × 4)	[31]
Immune Checkpoint Inhibition: Anti-Podoplanin monoclonal antibody	Phase I/II clinical trial of three dogs with oral melanoma (stage I, stage III, and stage IV) with endogenously expressed canine podoplanin	SD: 1 case (stage I) PD: 2 cases (stage III, and stage IV) No severe adverse effects were observed.	[33]
Immune Checkpoint Inhibition: Anti-canine PD-1 monoclonal antibody	Pre-clinical study of 21 cases of OMM: 4 cases of Stage III and 17 cases of Stage IV	PR: 2 cases (Stage IV) SD: 5 cases (Stage IV) PD: 14 cases (Stage III × 4, Stage IV × 10)	[32]
CAR-TILs targeting HER2	Pre-clinical trial of two dogs with HER2-positive aggressive melanoma. One dog with OMM (stage IV) and one dog with subungual melanoma (stage IV)	The dog with OMM experienced PD. The dog with subungual melanoma did not experience tumor recurrence within the follow-up period of the study and maintained a tumor-free status one-year post-treatment.	[37]

PR: partial response—a ≥50% reduction in tumor volume with no new tumors; SD: Stable disease—a <50% change in tumor volume and no new tumors; PD: progressive disease—a ≥50% increase in tumor volume or the development of new tumors; CAR-T: Chimeric antigen receptor T-cell immunotherapy; CAR-TILs: chimeric antigen receptor tumor-infiltrating lymphocytes; HER2: human epidermal growth factor receptor 2.

**Table 3:** Summary of small molecule signaling inhibitors’ effectiveness in canine oral malignant melanoma.

Inhibitor and Mechanism	Study Subject	Response Rate	Ref.
Rapamycin: Target cell signaling pathways mTOR	12, 23, and 50	1. Decreases in phosphorylated mTOR expression and phosphorylated p70S6K expression. 2. Dose-dependent decrease in surviving tumor cell fraction. Cell line 12: At 0.1 nM rapamycin, the surviving fraction was between 0.28 and 0.38. At 1 nM, it dropped to 0.1, and at 100 nM, it was 0.1. Cell line 23: At 0.1 nM, the surviving fraction was similar to cell.	[13]
AZD6244: Selectively non-ATP competitive inhibitor of MEK1/2. Combine with rapamycin: Inhibitor of mTOR	CML-10C2, CML-6 M, 17CM98, Jones, Parks	1. Canine melanoma cells exhibited sensitivity to AZD6244 and rapamycin. AZD6244 IC_50_ Values: Jones: 105 nM; 7CM98: 5.7 nM; CML-10C2: 391 nM; CML-6M: 42 nM Rapamycin IC_50_ Values: Jones: 2.6 nM; 17CM98: 0.118 nM; CML-10C2: 0.184 nM; CML-6M: 0.027 nM 2. The combination of AZD6244 and rapamycin demonstrated enhanced efficacy in reducing cell viability, evidenced by a greater reduction in IC_50_ values and more pronounced effects on inducing cell cycle arrest and apoptosis in melanoma cells.	[38]
GSK1120212: MEK inhibitor NVP-BEZ235: PI3K/mTOR inhibitor	UCDK9M1, UCDK9M2, UCDK9M3, UCDK9M4, UCDK9M5, Jones; Female nude mice with UCDK9M5 xenografts	1. The drug combination synergistically reduced cell survival by activating caspase 3/7, altering cell cycle and Bcl-2 protein expression and inducing apoptosis. 2. *In vivo*, the drug combination targeted signaling pathways, enhancing the reduction of mediators p-ERK, p-AKT, p-S6, and 4E-BP1.	[22]
Trametibin: MEK inhibitor Sapanisertib: Inhibits both mTORC1 and mTORC2 complexes	UCDK9M1, CDK9M2, UCDK9M3, CDK9M5, Jones; Nude mice with UCDK9M5 and UCDK9M1 xenografts	1. Trametinib IC_50_ ~ 10 nmol/L; 2. Sapanisertib IC_50_ 10 ~ 100 nmol/L; 3. The combined in vitro therapy enhanced cytotoxicity (produced apoptosis and altered the cell cycle). 4. The combined in vivo therapy limited primary mucosal melanoma xenograft growth in mice and tumor dissemination in a metastasis model.	[42]
Rapamycin and everolimus, inhibitors of mTOR, synergize with platinum chemotherapy	CML-1, CML-6M, CML-10c2, 17CM98	1. Rapamycin or everolimus combined with carboplatin showed a synergistic reduction in cell viability. 2. Phosphorylated mTOR levels were reduced by rapamycin and everolimus in all cell lines. 3. Both mTOR inhibitors decreased the extracellular acidification rate, indicating reduced glycolytic activity in melanoma cells.	[48]
LY3009120: Binding to all RAF isoforms, inhibiting their activity and suppressing downstream signaling	cRGO1, cRGO1.2, Mel1268, Mel0910, cRGO4, cRGO6	LY3009120 at the concentrations of 5 or 10 µM, cRGO1, cRGO1.2, cRGO4, cRGO6 Mel1268, and Mel0910 showed decreased cell proliferation by 90.58%, 80.09%, 94.82%, 93.52%, and 61.91%, respectively.	[24]
Toceranib: Tyrosine kinase inhibitor targeting KIT, PDGFR, VEGFR, and others	A dog with *KIT* mutation (*c.1725_1733del*)	The dog received toceranib orally every other day at a dosage of 2.6–2.9 mg/kg. Clinical signs such as halitosis, tumor hemorrhage, trismus, and facial edema improved, and the size of the metastatic lymph node reduced significantly by Day 15. The gingival tumor and associated masses in the masseter and pterygoid muscles decreased in size by Day 29 of treatment. However, toceranib treatment was terminated on Day 43 due to disease progression, and the dog died on Day 54.	[45]
Rivoceranib: VEGFR2 inhibitor	LMeC and LMeC cell-xenografted mice	1. Rivoceranib induces G0/G1 cell cycle arrest through the downregulation of cyclin-D1, thereby inhibiting cell cycle progression in LMeC; inhibits the mobility of canine tumor cell lines in vitro in a dose-dependent manner; suppresses tumor growth in xenograft mouse models. 2. Rivoceranib probably inhibited cyclin-D1 and downregulated VEGFR2 phosphorylation to reduce canine cell line viability *in vivo*. Rivoceranib showed apoptotic and anti-angiogenic activity *in vivo*.	[44]
BIO: Serine/threonine kinase inhibitors targeting GSK-3β.	CML-10C2, UCDK9M2, UCDK9M3	1. BIO treatment at 5 μM for 72 h enhanced β-catenin-mediated transcriptional activity, indicating GSK-3β inhibition. 2. Reduced cell proliferation and migration were observed.	[18]
MLN4924: NAE inhibitor interferes with the function of specific proteins by blocking neddylation system	CML-1, CML-6M, 17CM98, CML-10C2	1. MLN4924 efficiently decreases the viability of CMM cell lines CML-1, CML-6 M, and CML-10C2 *in vitro.* 2. Canine melanoma cell line 17CM98 showed relative insensitivity to MLN4924 treatment.	[47]

mTORC1: mammalian target of rapamycin complex 1; mTORC2: mammalian target of rapamycin complex 2; *KIT*: Proto-oncogene *c-KIT*; PDGFR: Platelet-Derived Growth Factor Receptor; VEGFR: Vascular Endothelial Growth Factor Receptor; BIO: 6-bromoindirubin-3′-oxime; GSK-3β: glycogen synthesis kinase-3β; NAE: NeDD8-activating enzyme; VEGFR2: Vascular Endothelial Growth Factor Receptor 2.

**Table 4:** Detailed information on the efficiency of indirect kinase inhibitors on canine melanoma based on in vitro cell lines and in vivo animals.

Protease/Kinase Inhibitor	Study Subject	Response Rate	Ref.
STA-1474	Phase I clinical trial (dog with OMM)	During the 4th STA-1474 treatment cycle, extravasation altered drug pharmacokinetics. A significant oral mass reduction was observed after 7 days, and a CT scan confirmed a partial response.	[50]
BCH and LPM	Cell lines (CMeC-1, CMeC-2, LMeC, PuMeC, KmeC)	1. BCH and LPM inhibited cell growth of CMeC-1 in a dose-dependent manner. IC_50_ values for BCH or LPM were 43 ± 3.9 mM and 1.7 ± 0.19 μM, respectively. 2. LAT1 inhibitors enhance the inhibitory activities for cell growth produced by conventional anti-cancer drugs.	[53]
Verdinexor (KPT-335)	Cell lines (Mel 23, Mel 36, Mel 69, Mel 83)	1. KPT-335 inhibited proliferation, blocked colony formation, and induced apoptosis at physiologically relevant drug concentrations. 2. Downregulation of the XPO1 protein while inducing an increase in XPO1 mRNA. 3. Upregulated expression of tumor suppressor proteins p53 and p21, promoting their nuclear localization.	[23]
Verdinexor (KPT-335)	Cell line (323610-3)	SINE compounds inhibited growth and induced apoptosis against melanoma cell lines, as evidenced by a 70 nM IC_50_ value for the 323610-3 melanoma cell line.	[15]
Oligomycin A (F1Fo ATPase inhibitor)	Cell lines (CMM-1, CMM-2, ChMC, NIP, NML, OMJ)	Oligomycin A showed potent cell growth inhibition activity against CMM-1, ChMC, and NIP, with IC_50_ values of 5.3 × 10^−4^–1.7 × 10^−3^ μM; NML, CMM-2, and OMJ were resistant to oligomycin A, with IC_50_ values of 5.0–10.5 μM.	[16]
HDIs	Cell lines (UCDK9M2, UCDK9M4)	1. JARID1-targeted HDIs significantly reduced cell survival without affecting DNA damage repair kinetics and were effective against cisplatin-resistant cell lines. 2. HDIs delay the resolution of DNA damage markers, such as p53BP1 and phosphorylated γ-H2AX, suggesting a delay in DNA repair kinetics.	[51]
ACT1 and BBI	Cell line (TLM1)	1. ACT1 alone did not significantly decrease cell viability. 2. BBI alone significantly decreased cell viability at a concentration of 400 µg/mL. 3. The combined treatment of aCT1 (200 µM) and BBI (400 µg/mL) significantly decreased cell viability more than either treatment alone.	[54]

STA-1474: precursor of ganetespib; LAT1: L-type amino acid transporter 1; BCH: 2-amino-2-norbornane-carboxylic acid; LPM: melphalan; XPO1: nuclear export protein exportin 1; SINE: selective inhibitor of nuclear exports; HDIs: histone demethylase inhibitors; JARID1: a histone H3 demethylase implicated in proliferation-dormancy regulation and drug sensitivity in human cancers; aCT1: alpha-connexin carboxyl-terminal peptide; BBI: Bowman-Birk protease inhibitor.

**Table 5:** Detailed information on the efficiency of other targeted therapies on canine melanoma based on in vitro cell lines and in vivo models.

Drug	Study Subject	Response Rate	Ref.
cAT-MSC-IFN-β	Cell line (LMeC) and BALB/c nude mouse xenografts	1. *In vitro* efficiency: cAT-MSC-IFN-β can directly inhibit the growth of LMeC as compared to control (67.76% of control growth, *p* < 0.05). 2. LMeC cells co-cultured with cAT-MSC-IFN-β showed increases in the G0/G1 phase of the cell cycle compared to the controls (*p* < 0.01). G1 arrest occurred concurrently with a reduction in the percentage of S phase cells (*p* < 0.01 and *p* < 0.001). 3. *In vivo* efficiency: In BALB/c nude mouse xenografts with LMeC cells, cAT-MSC-IFN-β and low-dose cisplatin significantly reduced tumor volume compared to other groups. TUNEL assay confirmed apoptosis at the tumor site, showing the combination induced cell death. 4. Homing of cAT-MSC-IFN-β: Fluorescent microscopy analysis showed homing of cAT-MSC-IFN-β to the tumor site, implying that these modified stem cells were able to target the tumor microenvironment effectively.	[60]
Hyaluronidase-armed canine oncolytic adenovirus	Cell lines (17CM98, CML1) and CML1 xenograft model in nude mice	*In vivo* experiment: intratumoral treatment with ICOCAV17 reduced tumor volume five-fold compared to the PBS control and 2.7-fold compared to CAV2 at the end of the experiment, with prolonged survival.	[58]
Bacillus anthracis (anthrax) toxin	Five dogs with spontaneous OMM	1. *In vivo:* no disease progression; 4 dogs showed tumor reduction varying from 12% to 63%. One dog showed a 20% increase in the tumor due to local edema. 2. Histopathology showed necrosis of tumor cells and blood vessel walls after treatment. 3. No significant systemic side effects were noted.	[67]
microRNA-205BP/S3	Ten dogs with CMM: stage I × 2; stage II × 4; stage III × 3; stage IV × 1	CR: 5/10 (I × 1, II × 3, III × 1) SD: 3/10 (I × 1, III × 2) PD: 2/10 (II × 1, IV × 1) No side effects were observed. Median overall survival: 340 days (n = 7). Median progression-free survival: 105 days (n = 6).	[62]
microRNA-634	Cell lines (KMeC, CMM1, CMeC-1)	1. miR-205 in canine melanoma cells heightened their radiation sensitivity, an effect linked to the suppressed activity of E2 transcription factor 1 (E2F1)-ATM signaling, which was confirmed by E2F1 knockdown and ATM knockout in the cell lines. 2. miR-205 overexpression led to a decrease in the expression of E2F1 and ATM, which are involved in the DNA damage response pathway.	[64]
microRNA-634	Seven dogs with spontaneous CMM	1. PR: 3 lesions; SD: 2 lesions; PD: 3 lesions. 2. The median treatment period was 53 d (range 23-652 d). miRNA was administered a median of seven times (range 4-76 times) at 2-10 nmol per dose. 3. Local administration to lung metastasis under ultrasound guidance induced tumor shrinkage.	[63]
Nonsteroidal Anti-Inflammatory Drugs	Cell lines (KMeC, LMeC, Mi, Pu, C1, C2, CMM7, CMM8, CMM9, MM10, CMM11, CMM12)	1. NSAIDs exhibited anti-tumor effects only at high concentrations, suggesting their action might be mediated through COX/PG-independent pathways. 2. There was no strong correlation between the sensitivity to NSAIDs and the expression of COX-related molecules. 3. Transcriptome analysis of a melanoma cell line exposed to NSAIDs identified novel candidate genes potentially involved in the anti-tumor effects of NSAIDs, indicating that these effects might be mediated through mechanisms independent of COX inhibition.	[20]
Atorvastatin	Cell lines (YCC, ITP, HTR)	1. Atorvastatin could potentially inhibit the growth of melanoma cells, especially those with high ZEB expression and mesenchymal-like properties. 2. The IC_50_ values for melanoma cell lines treated with atorvastatin ranged from 5.92 to 9.56 μM at 48 h, indicating a varying level of sensitivity among the melanoma cell lines tested.	[21]

cAT-MSC-IFN-β: Adipose Tissue-Derived Mesenchymal Stem Cells Expressing Interferon-β; CR: complete response, complete resolution of measurable tumor; COX: cyclooxygenase; PG: prostaglandins.

## Supplementary Materials

The following supporting information can be downloaded at https://www.mdpi.com/article/10.3390/ijms251910387/s1.

## References

[1] Gillard M., Cadieu E., De Brito C., Abadie J., Vergier B., Devauchelle P., Degorce F., Dréano S., Primot A., Dorso L. (2014). Naturally occurring melanomas in dogs as models for non-UV pathways of human melanomas. Pigment. Cell Melanoma Res..

[2] Prouteau A., André C. (2019). Canine Melanomas as Models for Human Melanomas: Clinical, Histological, and Genetic Comparison. Genes.

[3] Simpson R.M., Bastian B.C., Michael H.T., Webster J.D., Prasad M.L., Conway C.M., Prieto V.M., Gary J.M., Goldschmidt M.H., Esplin D.G. (2014). Sporadic naturally occurring melanoma in dogs as a preclinical model for human melanoma. Pigment. Cell Melanoma Res..

[4] van der Weyden L., Brenn T., Patton E.E., Wood G.A., Adams D.J. (2020). Spontaneously occurring melanoma in animals and their relevance to human melanoma. J. Pathol..

[5] Bongiovanni L., Brachelente C., Dow S., Bergman P.J. (2022). Editorial: Canine melanoma in comparative oncology: Translate research advances to develop new diagnostic and therapeutic options. Front. Vet. Sci..

[6] Hernández I.B., Kromhout J.Z., Teske E., Hennink W.E., van Nimwegen S.A., Oliveira S. (2021). Molecular targets for anticancer therapies in companion animals and humans: What can we learn from each other?. Theranostics.

[7] Hardwick L. (2021). A Comparative View on Molecular Alterations and Potential Therapeutic Strategies for Canine Oral Melanoma. Vet. Sci..

[8] Regan D., Guth A., Coy J., Dow S. (2016). Cancer immunotherapy in veterinary medicine: Current options and new developments. Vet. J..

[9] Maeda S. (2023). Second era of molecular-targeted cancer therapies in dogs. J. Vet. Med. Sci..

[10] Modiano J.F., Ritt M.G., Wojcieszyn J. (1999). The Molecular Basis of Canine Melanoma: Pathogenesis and Trends in Diagnosis and Therapy. J. Vet. Intern. Med..

[11] Hernandez B., Adissu H.A., Wei B.-R., Michael H.T., Merlino G., Simpson R.M. (2018). Naturally Occurring Canine Melanoma as a Predictive Comparative Oncology Model for Human Mucosal and Other Triple Wild-Type Melanomas. Int. J. Mol. Sci..

[12] Slim K., Nini E., Forestier D., Kwiatkowski F., Panis Y., Chipponi J. (2003). Methodological index for non-randomized studies (minors): Development and validation of a new instrument. ANZ J. Surg..

[13] Kent M.S., Collins C.J., Ye F. (2009). Activation of the AKT and mammalian target of rapamycin pathways and the inhibitory effects of rapamycin on those pathways in canine malignant melanoma cell lines. Am. J. Vet. Res..

[14] Alexander A., Huelsmeyer M., Mitzey A., Dubielzig R., Kurzman I., MacEwen E., Vail D. (2006). Development of an allogeneic whole-cell tumor vaccine expressing xenogeneic gp100 and its implementation in a phase II clinical trial in canine patients with malignant melanoma. Cancer Immunol. Immunother..

[15] London C.A., Bernabe L.F., Barnard S., Kisseberth W.C., Borgatti A., Henson M., Wilson H., Jensen K., Ito D., Modiano J.F. (2014). Preclinical Evaluation of the Novel, Orally Bioavailable Selective Inhibitor of Nuclear Export (SINE) KPT-335 in Spontaneous Canine Cancer: Results of a Phase I Study. PLoS ONE.

[16] Kuroki S., Kobayashi M., Tani H., Miyamoto R., Kurita S., Tamura K., Ono K., Washizu T., Bonkobara M. (2017). Selective growth inhibition by suppression of F1Fo ATPase in canine malignant melanoma cell lines. J. Vet. Pharmacol. Ther..

[17] Inoue K., Ohashi E., Kadosawa T., Hong S.-H., Matsunaga S., Mochizuki M., Nishimura R., Sasaki N. (2004). Establishment and Characterization of Four Canine Melanoma Cell Lines. J. Vet. Med. Sci..

[18] Chon E., Flanagan B., Rodrigues L.C.d.S., Piskun C., Stein T.J. (2015). 6-Bromoindirubin-3′oxime (BIO) decreases proliferation and migration of canine melanoma cell lines. Vet. J..

[19] Ohashi E., Hong S.-H., Takahashi T., Nakagawa T., Mochizuki M., Nishimura R., Sasaki N. (2001). Effect of retinoids on growth inhibition of two canine melanoma cell lines. J. Vet. Med. Sci..

[20] Yoshitake R., Saeki K., Watanabe M., Nakaoka N., Ong S., Hanafusa M., Choisunirachon N., Fujita N., Nishimura R., Nakagawa T. (2017). Molecular investigation of the direct anti-tumour effects of nonsteroidal anti-inflammatory drugs in a panel of canine cancer cell lines. Vet. J..

[21] Ishikawa T., Osaki T., Sugiura A., Tashiro J., Warita T., Hosaka Y.Z., Warita K. (2022). Atorvastatin preferentially inhibits the growth of high ZEB-expressing canine cancer cells. Vet. Comp. Oncol..

[22] Wei B., Michael H.T., Halsey C.H., Peer C.J., Adhikari A., Dwyer J.E., Hoover S.B., El Meskini R., Kozlov S., Ohler Z.W. (2016). Synergistic targeted inhibition of MEK and dual PI3K/mTOR diminishes viability and inhibits tumor growth of canine melanoma underscoring its utility as a preclinical model for human mucosal melanoma. Pigment. Cell Melanoma Res..

[23] Breit M.N., Kisseberth W.C., Bear M.D., Landesman Y., Kashyap T., McCauley D., Kauffman M.G., Shacham S., London C.A. (2014). Biologic activity of the novel orally bioavailable selective inhibitor of nuclear export (SINE) KPT-335 against canine melanoma cell lines. BMC Vet. Res..

[24] Gao Y., Packeiser E.M., Wendt S., Sekora A., Cavalleri J.M.V., Pratscher B., Alammar M., Hühns M., Brenig B., Junghanss C. (2024). Cross-Species Comparison of the Pan-RAF Inhibitor LY3009120’s Anti-Tumor Effects in Equine, Canine, and Human Malignant Melanoma Cell Lines. Genes.

[25] Atherton M.J., Morris J.S., McDermott M.R., Lichty B.D. (2016). Cancer immunology and canine malignant melanoma: A comparative review. Vet. Immunol. Immunopathol..

[26] Adams G.P., Weiner L.M. (2005). Monoclonal antibody therapy of cancer. Nat. Biotechnol..

[27] Helfand S.C., Soergel S.A., Donner R.L., Gan J., Hank J.A., Lindstrom M.J., Sondel P.M. (1994). Potential to involve multiple effector cells with human recombinant interleukin-2 and antiganglioside monoclonal antibodies in a canine malignant melanoma immunotherapy model. J. Immunother. Emphas. Tumor. Immunol..

[28] Rosenberg S.A., Yang J.C., Topalian S.L., Schwartzentruber D.J., Weber J.S., Parkinson D.R., Seipp C.A., Einhorn J.H., White D.E. (1994). Treatment of 283 consecutive patients with metastatic melanoma or renal cell cancer using high-dose bolus interleukin 2. JAMA.

[29] Alsaab H.O., Sau S., Alzhrani R., Tatiparti K., Bhise K., Kashaw S.K., Iyer A.K. (2017). PD-1 and PD-L1 Checkpoint Signaling Inhibition for Cancer Immunotherapy: Mechanism, Combinations, and Clinical Outcome. Front. Pharmacol..

[30] Lee H.T., Lee J.Y., Lim H., Lee S.H., Moon Y.J., Pyo H.J., Ryu S.E., Shin W., Heo Y.S. (2017). Molecular mechanism of PD-1/PD-L1 blockade via anti-PD-L1 antibodies atezolizumab and durvalumab. Sci. Rep..

[31] Maekawa N., Konnai S., Takagi S., Kagawa Y., Okagawa T., Nishimori A., Ikebuchi R., Izumi Y., Deguchi T., Nakajima C. (2017). A canine chimeric monoclonal antibody targeting PD-L1 and its clinical efficacy in canine oral malignant melanoma or undifferentiated sarcoma. Sci. Rep..

[32] Igase M., Nemoto Y., Itamoto K., Tani K., Nakaichi M., Sakurai M., Sakai Y., Noguchi S., Kato M., Tsukui T. (2020). A pilot clinical study of the therapeutic antibody against canine PD-1 for advanced spontaneous cancers in dogs. Sci. Rep..

[33] Kamoto S., Shinada M., Kato D., Yoshimoto S., Ikeda N., Tsuboi M., Yoshitake R., Eto S., Hashimoto Y., Takahashi Y. (2020). Phase I/II Clinical Trial of the Anti-Podoplanin Monoclonal Antibody Therapy in Dogs with Malignant Melanoma. Cells.

[34] Chen S., Zhang Z., Zheng X., Tao H., Zhang S., Ma J., Liu Z., Wang J., Qian Y., Cui P. (2021). Response Efficacy of PD-1 and PD-L1 Inhibitors in Clinical Trials: A Systematic Review and Meta-Analysis. Front. Oncol..

[35] Maalej K.M., Merhi M., Inchakalody V.P., Mestiri S., Alam M., Maccalli C., Cherif H., Uddin S., Steinhoff M., Marincola F.M. (2023). CAR-cell therapy in the era of solid tumor treatment: Current challenges and emerging therapeutic advances. Mol. Cancer.

[36] Dana H., Chalbatani G.M., Jalali S.A., Mirzaei H.R., Grupp S.A., Suarez E.R., Rapôso C., Webster T.J. (2021). CAR-T cells: Early successes in blood cancer and challenges in solid tumors. Acta Pharm. Sin. B.

[37] Forsberg E.M.V., Riise R., Saellström S., Karlsson J., Alsén S., Bucher V., Hemminki A.E., Bagge R.O., Ny L., Nilsson L.M. (2023). Treatment with Anti-HER2 Chimeric Antigen Receptor Tumor-Infiltrating Lymphocytes (CAR-TILs) Is Safe and Associated with Antitumor Efficacy in Mice and Companion Dogs. Cancers.

[38] Fowles J.S., Denton C.L., Gustafson D.L. (2015). Comparative analysis of MAPK and PI3K/AKT pathway activation and inhibition in human and canine melanoma: Pathway activation and inhibition in human and canine melanoma. Vet. Comp. Oncol..

[39] Meier F., Busch S., Lasithiotakis K., Kulms D., Garbe C., Maczey E., Herlyn M., Schittek B. (2007). Combined targeting of MAPK and AKT signalling pathways is a promising strategy for melanoma treatment. Br. J. Dermatol..

[40] Chen X., Liu F., Song X., Wang Z., Dong Z., Hu Z., Lan R., Guan W., Zhou T., Xu X. (2010). Rapamycin regulates Akt and ERK phosphorylation through mTORC1 and mTORC2 signaling pathways. Mol. Carcinog..

[41] Zeiser R., Andrlová H., Meiss F. (2018). Trametinib (GSK1120212). Recent Results Cancer Res..

[42] Wei B.R., Hoover S.B., Peer C.J., Dwyer J.E., Adissu H.A., Shankarappa P., Yang H., Lee M., Peat T.J., Figg W.D. (2020). Efficacy, Tolerability, and Pharmacokinetics of Combined Targeted MEK and Dual mTORC1/2 Inhibition in a Preclinical Model of Mucosal Melanoma. Mol. Cancer Ther..

[43] Jang S., Strickland B., Finis L., Kooijman J.J., Melis J.J.T.M., Zaman G.J.R., Van Tornout J. (2023). Comparative biochemical kinase activity analysis identifies rivoceranib as a highly selective VEGFR2 inhibitor. Cancer Chemother. Pharmacol..

[44] Li Q., Kim Y.-S., An J.-H., Kwon J.-A., Han S.-H., Song W.-J., Youn H.-Y. (2021). Anti-tumor effects of rivoceranib against canine melanoma and mammary gland tumour in vitro and in vivo mouse xenograft models. BMC Vet. Res..

[45] Tani H., Miyamoto R., Noguchi S., Kurita S., Nagashima T., Michishita M., Yayoshi N., Tamura K., Bonkobara M. (2021). A canine case of malignant melanoma carrying a KIT c.1725_1733del mutation treated with toceranib: A case report and in vitro analysis. BMC Vet. Res..

[46] Brocca G., Poncina B., Sammarco A., Cavicchioli L., Castagnaro M. (2020). KIT Somatic Mutations and Immunohistochemical Expression in Canine Oral Melanoma. Animals.

[47] Wood E.A., Lu Z., Jia S., Assumpção A.L.F.V., Van Hesteren M.A., Huelsmeyer M.K., Vail D.M., Pan X. (2020). Pevonedistat targeted therapy inhibits canine melanoma cell growth through induction of DNA re-replication and senescence. Vet. Comp. Oncol..

[48] Bernard S., Poon A.C., Tam P.M., Mutsaers A.J. (2021). Investigation of the effects of mTOR inhibitors rapamycin and everolimus in combination with carboplatin on canine malignant melanoma cells. BMC Vet. Res..

[49] Youssef M.E., Cavalu S., Hasan A.M., Yahya G., Abd-Eldayem M.A., Saber S. (2023). Role of Ganetespib, an HSP90 Inhibitor, in Cancer Therapy: From Molecular Mechanisms to Clinical Practice. Int. J. Mol. Sci..

[50] London C.A., Bear M.D., McCleese J., Foley K.P., Paalangara R., Inoue T., Ying W., Barsoum J. (2011). Phase I evaluation of STA-1474, a prodrug of the novel HSP90 inhibitor ganetespib, in dogs with spontaneous cancer. PLoS ONE.

[51] Tobin S.J., Chang H., Kent M.S., Davies A.E. (2021). JARID1-targeted histone H3 demethylase inhibitors exhibit anti-proliferative activity and overcome cisplatin resistance in canine oral melanoma cell lines. Vet. Comp. Oncol..

[52] Wang N., Ma T., Yu B. (2023). Targeting epigenetic regulators to overcome drug resistance in cancers. Signal Transduct. Target. Ther..

[53] Fukumoto S., Hanazono K., Fu D.-R., Endo Y., Kadosawa T., Iwano H., Uchide T. (2013). A new treatment for human malignant melanoma targeting L-type amino acid transporter 1 (LAT1): A pilot study in a canine model. Biochem. Biophys. Res. Commun..

[54] Sato A., da Fonseca I.I.M., Nagamine M.K., de Toledo G.F., Olio R., Hernandez-Blazquez F.J., Yano T., Yeh E.S., Dagli M.L.Z. (2021). Effects of Alpha-Connexin Carboxyl-Terminal Peptide (aCT1) and Bowman-Birk Protease Inhibitor (BBI) on Canine Oral Mucosal Melanoma (OMM) Cells. Front. Vet. Sci..

[55] Rozanov D.V., Golubkov V.S., Strongin A.Y. (2005). Membrane type-1 matrix metalloproteinase (MT1-MMP) protects malignant cells from tumoricidal activity of re-engineered anthrax lethal toxin. Int. J. Biochem. Cell Biol..

[56] Nishiya A.T., Massoco C.O., Felizzola C.R., Perlmann E., Batschinski K., Tedardi M.V., Garcia J.S., Mendonça P.P., Teixeira T.F., Dagli M.L.Z. (2016). Comparative Aspects of Canine Melanoma. Vet. Sci..

[57] Cejalvo T., Perisé-Barrios A.J., del Portillo I., Laborda E., Rodriguez-Milla M.A., Cubillo I., Vázquez F., Sardón D., Ramirez M., Alemany R. (2018). Remission of Spontaneous Canine Tumors after Systemic Cellular Viroimmunotherapy. Cancer Res..

[58] Laborda E., Puig-Saus C., Rodriguez-García A., Moreno R., Cascalló M., Pastor J., Alemany R. (2014). A pRb-responsive, RGD-modified, and Hyaluronidase-armed Canine Oncolytic Adenovirus for Application in Veterinary Oncology. Mol. Ther..

[59] Bunnell B.A. (2021). Adipose Tissue-Derived Mesenchymal Stem Cells. Cells.

[60] Ahn J.O., Lee H.W., Seo K.W., Kang S.K., Ra J.C., Youn H.Y. (2013). Anti-tumor effect of adipose tissue derived-mesenchymal stem cells expressing interferon-β and treatment with cisplatin in a xenograft mouse model for canine melanoma. PLoS ONE.

[61] Sell M.C., Ramlogan-Steel C.A., Steel J.C., Dhungel B.P. (2023). MicroRNAs in cancer metastasis: Biological and therapeutic implications. Expert Rev. Mol. Med..

[62] Yoshikawa R., Mori T., Noguchi S., Akao Y., Maruo K., Kitade Y. (2019). Synthetic microRNA-205 exhibited tumour suppression in spontaneous canine malignant melanoma by intratumoral injection. Vet. Comp. Oncol..

[63] Yoshikawa R., Inoue J., Iwasaki R., Terauchi M., Fujii Y., Ohta M., Hasegawa T., Mizuno R., Mori T., Inazawa J. (2023). Therapeutic applications of local injection of hsa-miR-634 into canine spontaneous malignant melanoma tumors. Cancer Gene Ther..

[64] Wada Y., Noguchi S., Nishiyama Y., Matsuyama S., Mori T., Igase M., Mizuno T., Shimamura S., Shimada T. (2019). MicroRNA-205 enhances the radiosensitivity of canine oral melanoma cells by inhibiting E2F1. Jpn. J. Vet. Res..

[65] Sirtori C.R. (2014). The pharmacology of statins. Pharmacol. Res..

[66] Jiang W., Hu J.-W., He X.-R., Jin W.-L., He X.-Y. (2021). Statins: A repurposed drug to fight cancer. J. Exp. Clin. Cancer Res..

[67] Nishiya A.T., Nagamine M.K., da Fonseca I.I.M., Miraldo A.C., Scattone N.V., Guerra J.L., Xavier J.G., Santos M., Gomes C.O.M.d.S., Ward J.M. (2020). Inhibitory Effects of a Reengineered Anthrax Toxin on Canine Oral Mucosal Melanomas. Toxins.

[68] Stevenson V.B., Perry S.N., Todd M., Huckle W.R., LeRoith T. (2021). PD-1, PD-L1, and PD-L2 Gene Expression and Tumor Infiltrating Lymphocytes in Canine Melanoma. Vet. Pathol..

[69] Chan T., Yarchoan M., Jaffee E., Swanton C., Quezada S., Stenzinger A., Peters S. (2019). Development of tumor mutation burden as an immunotherapy biomarker: Utility for the oncology clinic. Ann. Oncol..

[70] Alsaihati B.A., Ho K.-L., Watson J., Feng Y., Wang T., Dobbin K.K., Zhao S. (2021). Canine tumor mutational burden is correlated with TP53 mutation across tumor types and breeds. Nat. Commun..

[71] Utsugi S., Ogihara K., Naya Y., Sunden Y., Nakamoto Y., Okamoto Y. (2022). Expression of L-type amino acid transporter 1 in canine and feline intracranial tumors. J. Vet. Med. Sci..

[72] Lisjak A., Lopes B.C., Pilla R., Nemec A., Suchodolski J.S., Tozon N. (2023). A Comparison of the Oral Microbiota in Healthy Dogs and Dogs with Oral Tumors. Animals.

[73] Kleber K.T., Iranpur K.R., Perry L.M., Cruz S.M., Razmara A.M., Culp W.T.N., Kent M.S., Eisen J.A., Rebhun R.B., Canter R.J. (2022). Using the canine microbiome to bridge translation of cancer immunotherapy from pre-clinical murine models to human clinical trials. Front. Immunol..

